# Multi-Omics Analysis Reveals the Potential Effects of Maternal Dietary Restriction on Fetal Muscle Growth and Development

**DOI:** 10.3390/nu15041051

**Published:** 2023-02-20

**Authors:** Xinyue Wang, Mingyu Shang, Wenping Hu, Li Zhang

**Affiliations:** Institute of Animal Sciences, Chinese Academy of Agricultural Sciences, Beijing 100193, China

**Keywords:** fetal muscle, maternal dietary restriction, sheep pregnancy model, multi-omics analysis, proteome, transcriptome

## Abstract

In terms of fetal muscle growth, development, and health, maternal nutrition is a crucial influence, although the exact biochemical mechanism by which this occurs is still not fully understood. To examine the potential impacts of maternal dietary restriction on fetal muscle development, the sheep maternal dietary restriction model was developed for this study. In our study, 12 pregnant ewes were evenly split into two experimental groups and fed either 75% or 100% of a maternal nutrient. In addition, a multi-omics analysis was used to study the embryonic longissimus dorsis on gestational days (GD) 85 and 135. The fetal weight at GD 135 was significantly below normal due to the maternal restricted diet (*p* < 0.01). When fetuses were exposed to the dietary deficit, 416 mRNAs and 40 proteins were significantly changed. At GD 85, the multi-omics analysis revealed that maternal dietary restriction led to a significant up-regulation of the cell cycle regulator *CDK2* gene in the cellular senescence signaling pathway, and the results of the qRT-PCR were similar to the multi-omics analysis, which showed that *SIX1*, *PAX7*, the cell cycle factors *CDK4* and *CDK6*, and the *BCL-2* apoptosis factor were up-regulated and several skeletal muscle marker genes, such as *MYF5* and *MyoD* were down-regulated. At GD 135, maternal dietary restriction blocks the muscle fiber differentiation and maturation. The multi-omics analysis revealed that the *TEAD1* gene was in the Hippo signaling pathway, the muscle marker genes *MYF5* and *MyoG* were significantly down-regulated, and the *TEAD1* binding of the down-regulated *VGLL3* gene might be potential mechanisms affecting myofiber differentiation and maturation. Knocking down the *CDK2* gene could inhibit the proliferation of primary embryonic myoblasts, and the expression levels of cell cycle regulatory factors *CDK4* and *CDK6* were significantly changed. Under low nutrient culture conditions, the number of myoblasts decreased and the expression of *CDK2*, *CDK6*, *MYF5*, *PAX7* and *BCL-2* changed, which was in perfect agreement with the multi-omics analysis. All of the findings from our study helped to clarify the potential effects of maternal dietary restriction on fetal muscle growth and development. They also provided a molecular foundation for understanding the molecular regulatory mechanisms of maternal nutrition on fetal muscle growth and development, as well as for the development of new medications and the management of related metabolic diseases.

## 1. Introduction

Maternal nutrition is a crucial intrauterine environmental component in embryonic growth and development, which is directly linked to the development of multiple organ systems and the advancement of many chronic metabolic illnesses [1]. Maternal dietary deficiency causes neural tube defects, cretinism, intrauterine growth retardation, preterm birth, endocrine dyscrasia, diabetes and organ maldevelopment [2,3,4,5,6]. Undernutrition is a significant global problem in newborns. Despite the efforts of many nations, organizations, such as the World Health Organization (WHO) and researchers, more research on the molecular mechanisms of maternal nutritional regulation of fetal skeletal muscle growth and development is still needed [7,8,9,10,11,12]. 

Pregnancy is a delicate and complex process. The growth and development of the fetus depends on the inner environment the mother provided. Extra pregnancy systems, such as an artificial and 3D cell culture uterus, have now been developed to study the tissue, but they still cannot replicate the regulation functions of maternal nutrition, hormones, the inner environment, and the genetic effect on fetus growth and development [13,14,15]. The metabolic and endocrine changes of sheep have been found to be more similar to those of humans than mice. Most sheep breeds are monotocous and have the appropriate size; therefore, sheep have been regarded as the best animal model for studying the intrauterine condition, fetal development, and newborn fate [16,17,18].

Meanwhile, our previous research indicates that as one of the key organs for nutritional requirements and metabolism, sheep embryonic skeletal muscle is influenced by energy metabolism [19]. Studies using animal models have shown that maternal nutrition affects human skeletal muscle development, phenotype and health [12,20,21,22]. Numerous studies have demonstrated that maternal dietary restriction affects fetal skeletal muscle growth, development, metabolism and insulin sensitivity [20,23,24], and changes the gene and protein expression in muscles, which results in muscle lipid content and growth of offspring [20], and reduces the number of muscle fibers and cross-sectional area. The potential reasons for this may be relative to the protein synthesis balance and metabolic disorders [25,26]. In the meantime, if lambs are born with appropriate or insufficient nourishment, it affects muscle growth, myofiber number and protein synthesis [27]. Several studies have shown that metabolism and insulin may affect muscle growth and development resulting in the loss of weight in skeletal muscle, changes in hormonal function [21,28] and the expression of the insulin-like growth factor gene [29,30]. These studies show that maternal nutritional deficits influence gene expression and metabolism in muscle cells, which affects muscle growth and development.

It has been demonstrated that the activity of *mTOR* may be the primary factor controlling the proliferation of myoblasts and the formation of secondary muscle fibers at the cellular level [31,32]. While maternal nutrition affects fetal growth and development, this is a complex biological process in which nutrition is the major factor that alters the expression of the fetal genome and may have lifelong consequences [1]. There are numerous attempts to explain the potential mechanism of how maternal dietary restriction affects fetal skeletal muscle growth and development [33,34,35,36,37]. Although high throughput technologies, such as proteome, transcriptome, methylomes, and multi-omics analysis, were used to reveal the potential regulation of molecular mechanisms, not all gene expressions of abnormal fetal skeletal muscle caused by maternal dietary restriction are fully analyzed [38,39,40]. Currently, the specific molecular mechanism underlying the changes in fetal skeletal muscle growth and development induced by maternal dietary deficiency remains unclear. Therefore, it is crucial to understand how changes in genes and proteins expression affect the molecular process by which maternal dietary restriction affects the growth and development of fetal skeletal muscle.

To better understand the mechanism of muscle response to dietary deficiency and the signal pathway modifications in skeletal muscle, a maternal dietary restriction sheep model was constructed in this study. A multi-omics profile analysis was conducted, and the findings emphasized the significance of the molecular mechanism by which maternal dietary restriction affects the growth and development of fetal muscles, and offered a fresh approach to the control of maternal and infant nutrition and illness management.

## 2. Materials and Methods

### 2.1. Animals, Diets, and Sample Collection

Adult Chinese merino sheep in good condition were randomly selected, and all sheep required supplementary feeding to achieve the same condition before breeding. For ewes, each of them was fed 0.6 kg/d of a pelleted diet, free forage (the ratio of hay to silage was 1:3), and water. Each of the rams was fed 0.8 kg/d of a pelleted diet, one egg, and 2 kg/d of carrot. At the same time, they were able to exercise appropriately. The quality of semen was checked every two days during the 15 days before breeding. Fifty multiparous ewes were estrus synchronized using a progesterone pessary, and after 12 days, their estrus rate was 93.7%. Then, we took the semen from the same ram three or four times in one day to breed with the ewes using artificial insemination.

**Figure 1:**
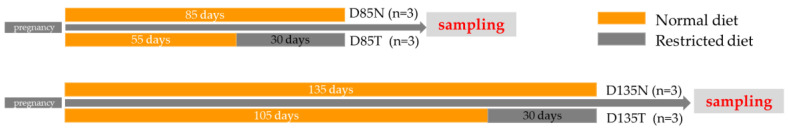
Dietary treatment and sampling diagram for the pregnant ewes. The long orange bar indicates the time of the maternal normal diet. The long gray bar indicates the time of the maternal restricted diets. D85N indicates the group of normal diets; D85T indicates the group of the maternal restricted diets. D135N indicates the group of normal diets; D135T indicates the group of restricted diets.

Studies showed that the formation and fusion of sheep myofibrils occurred around GD 50 and GD 80, respectively. At GD 100, the number of myofibrils was stable and they were largely mature at GD 135 [41]. In this study, we provided the normal and restricted diet to groups of 12 ewes on days 85 and 135 of pregnancy, respectively (D85N, D135N, D85T, D135T; N indicates normal diet, and T indicates restricted diet). Moreover, two comparable groups, such as D85T vs. D85N and D135T vs. D135N, were set for study. Animals were housed individually and assigned to one of two diets: normal diet (100%; *n* = 6); or restricted diet (75%; *n* = 6); based on the NZSAP for ewes on normal nutrition (Table S1). The details of the feeding method were as follows: A cesarean section was performed on six ewes on GD 85, before the operation, half of the ewes were randomly selected to undergo 30 days of maternal dietary restriction (D85T), while the other half received normal feeding (D85N). The cesarean section was performed on six ewes on GD 135, as well as the first experimental group, half of the ewes were randomly selected to undergo 30 days of maternal dietary restriction (D135T), while the other half received normal feeding (D135N) (Figure 1).

Meanwhile, diets were adjusted weekly based on individual body weights. Then, the fetuses with similar embryonic ages were taken out, and the growth trait phenotypes were measured. Based on reports [19], the longissimus dorsi were collected, and there were three biological repeats per time phase (no mixing between per time phase samples). Finally, these samples were immediately washed clear with PBS (pH 7.2) and then frozen in liquid nitrogen. All samples were kept at −80 °C until further analyses.

### 2.2. Phenotype Trait Analysis

The fetal body length, fetus weight, body height, fore-limb length, hind-limb length, liver weight, spleen weight, lungs weight and kidney weight from normal and restricted maternal diets were measured at GD 85 and 135, respectively. All data were analyzed by using Student’s 2-sided *t*-tests in SPSS 22.0 software (IBM Corp, Chicago, IL, USA).

### 2.3. mRNA Sequencing Analysis

Total RNA from 12 samples was extracted using Trizol reagent (Invitrogen, Pleasanton, CA, USA) following the manufacturer’s procedure. The total RNA quantity and purity were analyzed on the Bioanalyzer 2100 and the RNA 6000 Nano Lab Chip Kit (Agilent, Santa Clara, CA, USA) with a RIN number > 7.0. According to the manuscript of the Epicentre Ribo-Zero Gold Kit (Illumina, San Diego, CA, USA), approximately 10 ug of total RNA representing a specific adipose type was used to deprotease ribosomal RNA. Following purification, the poly(A)− or poly(A)+ RNA fractions were fragmented into small pieces using divalent cations at elevated temperatures. Then, the cleaved RNA fragments were reverse-transcribed to create the final cDNA library according to the protocol for the mRNA-Seq sample preparation kit (Illumina, San Diego, CA, USA), and the average insert size for the paired-end libraries was 300 bp (±50 bp). Then, we performed the paired-end sequencing on an Illumina HiSeq 4000 at LC-Bio, Hangzhou, China), following the vendor’s recommended protocol. We assembled the transcripts. Cutadapt was used to remove the reads that contained adaptor contamination, low-quality bases and undetermined bases. The sequence quality was verified using FastQC “http://www.bioinformatics.babraham.ac.uk/projects/fastqc/ (accessed on 19 September 2018) ”. We used Bowtie2 and Tophat2 to map the reads to the genome of the sheep. The mapped reads of each sample were assembled using StringTie. Then, samples were merged to reconstruct a comprehensive transcriptome using Perl scripts. Once the final transcriptome was generated, StringTie and Ballgown were used to estimate the expression levels of all mRNAs.

### 2.4. Proteome Quantitative Analysis

The total proteins from 12 fetal sheep were extracted, and the data preprocessing and TMT quantification methods were performed as we previously described [19].

### 2.5. Statistical Analysis

The data are presented as the means ± SE. Muscle growth and development relative mRNA abundance obtained from qPCR were analyzed using Student’s 2-sided *t*-tests in SPSS 22.0 software (IBM Corp, Chicago, IL, USA), with a significant difference threshold at *p* ≤ 0.05. For the transcriptome data, the gene abundance was compared using edgeR packages on a free online platform “http://www.omicshare.com/tools (accessed on 20 October 2018) ”, with a significant difference set at a false discovery rate (FDR) ≤ 0.05 and fold change ≥ 1. For the proteome data, significance was analyzed using Student’s *t*-tests; *p* ≤ 0.05 and fold change ≥ 1.5 indicated significance. The differential gene and protein expression data analyses were as previously described (X. Wang et al., 2020). We integrated the data from the mRNA sequencing and quantitative proteome analysis with a threshold at |log2| ≥ 1; *p* ≤ 0.05 and |ratio| ≥ 1.5; *p* ≤ 1, respectively, and analyzed the matched genes between mRNA and protein. Finally, the functional enrichment analysis of the differentially expressed genes identified the Gene Ontology (GO) terms, and Kyoto Encyclopedia of Genes and Genomes (KEGG) pathways was performed using the online resource DAVID (version 6.8) “https://david.ncifcrf.gov/ (accessed on 20 October 2018)”. The Wolfpsort v.0.2 “http://www.genscript.com/psort/wolf_psort.html (accessed on 20 October 2018)” software was used to perform subcellular structural localization prediction and classification statistics for differential genes.

### 2.6. qRT-PCR and PRM of mRNAs and Proteins in Muscle Tissues

Total RNA was isolated from the longissimus dorsi muscle (30–40 mg tissues) of 12 fetuses from each group with an RNA prep Pure Tissue Kit (Tiangen, Biotech Co. Ltd, Beijing, China). The maker genes for muscle growth and development were detected via quantitative real-time PCR (qRT-PCR), and the β-actin was used as the reference primer. Primers were designed for the selected mRNAs from the transcriptome database (Table S2). Real-time PCR was performed using the TB Green Premix Ex Taq TM Ⅱ (Takara, Dalian, China) on the 7500 Real-time System (Applied Biosystems, Waltham, MA, USA). The relative expression of the mRNA was calculated using the 2^−ΔΔCt^ method. 

In our study, the TMT quantitation results were validated using the PRM, and the details of the PRM methods were performed, as we previously described [19]. Twenty differential abundance proteins were randomly selected from D135T vs. D135N (Table S3).

### 2.7. Knock-Down CDK2 Gene Expression in the Sheep Primary Embryonic Myoblasts

According to the design principle of siRNA, three pairs of *CDK2* siRNAs and negative control siRNAs were successfully designed and synthesized (Table S4). The sheep primary embryonic myoblasts were seeded on 12 well plates. On the second day, the medium was changed to Opti-DMEM/F12 (Gibco, Grand Island, NE, USA), and three *CDK2* siRNAs and negative control siRNA (siNC) (30 ng/well) were transfected with lipofectamine 3000 agent (Invitrogen, Pleasanton, CA, USA), and the untreated group was used as the control. Then, after 6 h, Opti-DMEM/F12 (Gibco, Grand Island, NE, USA) containing 10% FBS was used to replace the medium. Then, after 24 h of Opti-DMEM/F12 (Gibco, Grand Island, NE, USA) culture with 10% FBS, the cells were washed twice with cold PBS, and then the total RNA was extracted from the cells with TRIZOL (Invitrogen, Pleasanton, CA, USA), and the cDNA samples were obtained according to the instructions of Prime Script RT reagent Kit with gDNA Eraser (Takara, Dalian, China). Finally, qRT-PCR was used to measure the knock-down efficiency of three *CDK2* siRNAs and related gene expressions.

### 2.8. Gene and Phenotypic Analysis of Sheep Primary Embryonic Myoblasts Cultured under Different Nutrient Conditions

Equal amounts of sheep primary myoblasts were evenly spread in 6-well plates and cultured using Opti-DMEM/F12 (Gibco, Grand Island, NE, USA) containing 10% FBS until the cell fusion was 80–90%, when three fields of view were randomly found for photographing, and normal and restricted nutrition culture groups were set up with three biological replicates in each group, respectively. The cells in the normal nutrition group were replaced with Opti-DMEM/F12 (Gibco, Grand Island, NE, USA) containing 10% FBS to continue the culture, and the cells in the restricted nutrition group were replaced with Opti-DMEM/F12 (Gibco, Grand Island, NE, USA) for culture. The cells in both groups were randomly found in three fields of view at 12 h, 24 h and 48 h after changing the medium for photography. At the same time, RNA was extracted using TRIZOL (Invitrogen, Pleasanton, CA, USA) at 48 h in the normal and restricted nutrient groups, and the cDNA samples were obtained according to the instructions of the Prime Script RT reagent Kit With gDNA Eraser (Takara, Dalian, China). Finally, the related genes were quantified by qRT-PCR.

## 3. Results

### 3.1. Maternal Dietary Restriction Nutrition Causes the Fetal Body Weight to Decrease Significantly 

**Figure 2:**
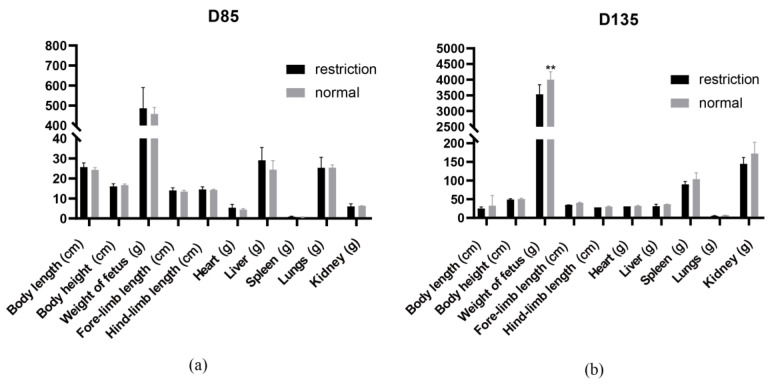
Phenotype analysis. Phenotype analysis of the fetal sheep at (**a**) GD 85 and (**b**) GD 135. The black box is the restricted maternal nutrition, the gray box is the normal maternal nutrition, and *p* < 0.01 (**) indicates an extremely significant difference.

The body length, fetus weight, body height, fore-limb length, hind-limb length, liver weight, spleen weight, lung weight and kidney weight of fetuses from the normal and restricted maternal diets were measured at the GD 85 and 135. The results indicated that at GD 135, the fetal body weight under the maternal restriction nutrition was significantly lower than that in the maternal normal nutrition group (*p* ≤ 0.01) (Figure 2).

### 3.2. mRNA and Protein Expression Profiles

To assess the differentially expressed genes involved in the two maternal diet patterns, fetal longissimus dorsi were collected from GD 85 and 135 in sheep fetuses for the transcriptome profiling of all mRNAs via high-throughput sequencing. For the RNA library (without ribosomal), an average of 88,000,000 valid reads were obtained from the per-sample tests. All 12 samples had at least 97% reads exceeding Q30, and 82% of these reads were uniquely aligned to the reference sheep genome. A total of 47,270 transcripts, including 22,606 genes were obtained. Moreover, 21,380 and 43,097 different expression genes and transcripts were identified, respectively. Meanwhile, the principal component analysis (PCA) was used for estimating the repeatability of all transcripts, and all results showed that the repeatability was ideal. Given the PCA results, all samples were clearly distinguished into two major categories (Figure S1a). Analyzing all transcripts, a total of 161 and 255 differential expressions (DE)-mRNAs were significantly expressed in D85T vs. D85N and D135T vs. D135N, with 85 DE-mRNAs up-regulated and 76 down-regulated in D85T vs. D85N, and in D135T vs. D135N, with 101 DE-mRNAs up-regulated and 154 down-regulated (Figure S1b). 

Additionally, the protein profile was analyzed, as in our previous article [19], and in this study, there were a total of 40 differentially abundant proteins in D85T vs. D85N and D135T vs. D135N. TMT protein quantification of embryonic skeletal muscle samples from maternal dietary restriction and normal groups (three replicates for each developmental stage) at GD 85 and 135 were compared and analyzed. The results showed that 10 differentially abundant proteins in the D85T vs. D85N, in which seven differentially abundant proteins were up-regulated and three differentially abundant proteins were down-regulated. There were 30 differentially abundant proteins in the D135T vs. D135N, in which 10 differentially abundant proteins were up-regulated and 20 differentially abundant proteins were down-regulated (Figure S1d). Therefore, based on the results of the PCA, all samples were clearly distinguished into two major categories (Figure S1c).

### 3.3. Integrating the mRNA and Protein Expression Profile Analyses

Furthermore, a correlation analysis of the mRNA and protein, according to the requirements (Figure S1e) and the integrated analysis showed that 3030 mRNA-protein pairs were obtained in two comparable groups. Within these two groups, 47 differentially expressed mRNA-protein pairs (DE genes) were significantly expressed in D85T vs. D85N, including eight up-up regulated mRNA-protein pairs, 13 down-down regulated mRNA-protein pairs, 11 down-up regulated pairs, and 15 up-down regulated pairs. Additionally, 50 mRNA-protein pairs were significantly expressed in D135T vs. D135N, including 14 mRNA-protein pairs that were up-up regulated, 15 mRNA-protein pairs that were down-down regulated, 11 mRNA-protein pairs that were down-up regulated, and 10 mRNA-protein pairs that were up-down regulated in this comparable group (Table S5).

### 3.4. At GD 85, Maternal Dietary Restriction Alters the Cell Cycle of Sheep Embryonic Myoblasts 

#### 3.4.1. At the mRNA and Protein Levels, the Cell Cycle and Apoptosis-Related Gene Expression Changes

**Figure 3:**
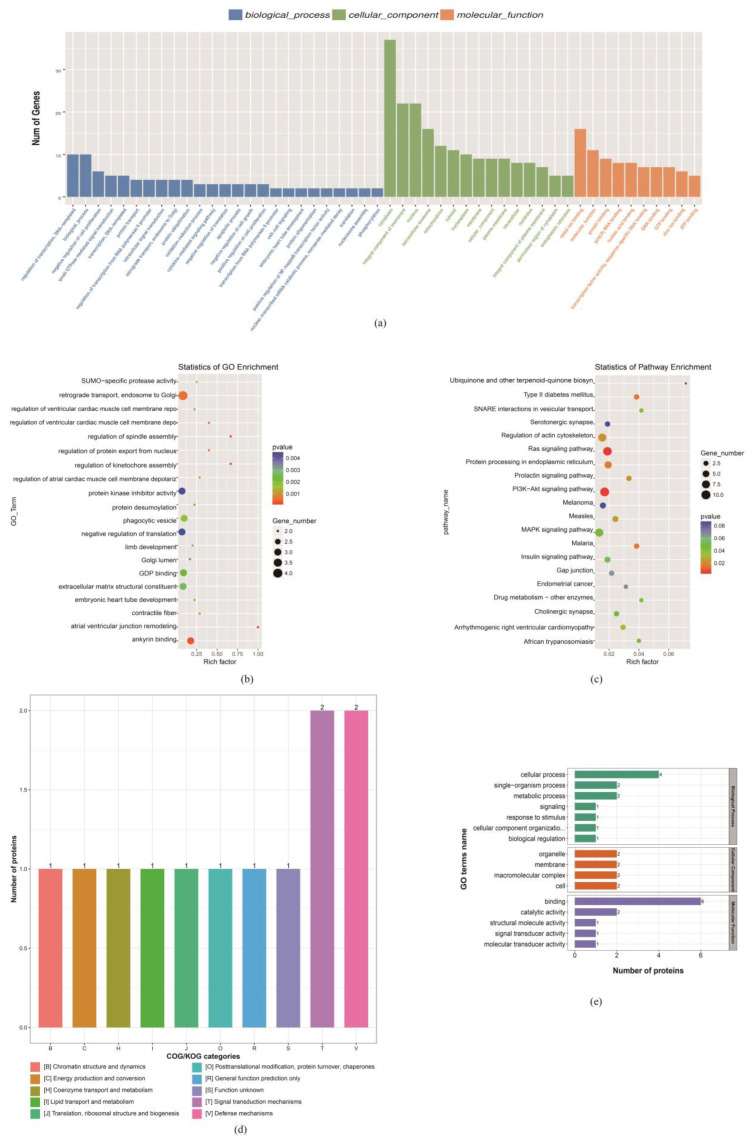
D85T vs. D85N differential expression mRNA and protein enrichment analysis. (**a**) Differential expression mRNA Gene Ontology (GO) term analysis; mRNAs, as classified into three main categories by the GO analysis, blue indicates the biological process, green indicates the cellular component and orange indicates the molecular function. (**b**) GO enrichment analysis. (**c**) Differential expression mRNA KEGG analysis, in (**b**,**c**), the size and color of the bubble indicate the number and significant characters of the differential expression mRNAs that are enriched in GO terms. (**d**) In the functional classification chart of the differential protein abundance (COG), the orthologous proteins are classified into different functions. (**e**) Differential protein abundance GO term analysis. Proteins, as classified into three main categories by the GO analysis, green, indicates a biological process, orange indicates the cellular component and purple indicates molecular function.

First, the GO analysis was performed with the mRNA functional categories, including biological processes (BPs), cellular components (CCs), and molecular functions (MFs) for D85T vs. D85N DE-mRNA, respectively. Figure 3a shows that the annotated GO terms of DE-mRNA in the maternal dietary restriction comparable group covered negative regulation of the cell proliferation, protein ubiquitination, the negative regulation of the signal translation, apoptosis process and the negative regulation of cell growth as major subcategories of the BPs; cytoplasm, integral component of the membrane, nucleus, extracellular exosome and mitochondrion were the key subcategories of the CCs; metal-ion binding and protein binding poly(A) RNA binding were the pivotal MF subcategories. The GO enrichment analysis indicated that the GO terms with the function of the cell cycle, metabolism, signal translation and fetal development were significantly enriched in this phase, such as the regulation of the spindle assembly, regulation of the kinetochore assembly, GDP binding, limb development, protein kinase inhibitor activity and negative regulation of translation (Figure 3b). Furthermore, the results of the KEGG enrichment showed that the PI3K-AKT signal pathways were significantly enriched, and the gene with the functions of cell cycle regulation and negative regulation of cell proliferation, *ING2*, was a potential key regulatory factor with significant enrichment in the PI3K-AKT signal pathways (Figure 3c).

Then, the differentially abundant proteins (DAPs) in D85T vs. D85N were analyzed using the methods of the functional classification chart of the differential protein abundance (COG) and gene ontology (GO) analyses, as Figure 3d shows that the DAPs were mainly enriched in major function classifications, such as lipid transport and metabolism, coenzyme transport and metabolism, energy transport and metabolism, translation, ribosomal structure, biogenesis and chromatin structure and dynamics, and these DAPs were significantly enriched in 16 GO terms that play a molecular binding role in cellular biological processes (Figure 3e). Meanwhile, the FAU, which regulates cell proliferation and apoptosis, was significantly up-regulated in this comparison group, but the DAPs were not enriched in any signaling pathway by the KEGG enrichment analysis.

#### 3.4.2. *CDK2* May Play a Key Role in Myoblast Cell Cycle Changes in Response to Maternal Dietary Deficiency

**Figure 4:**
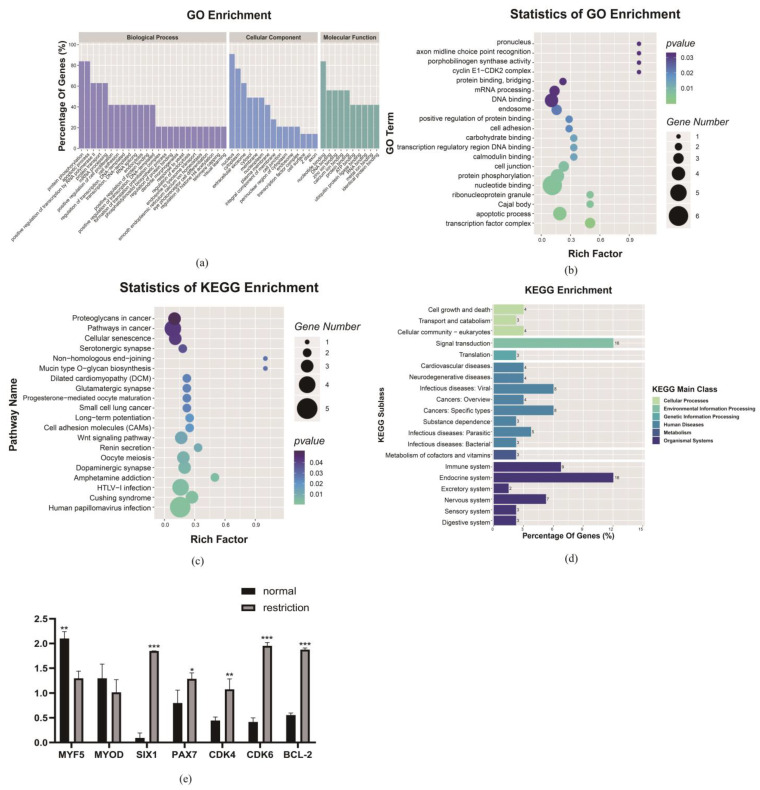
D85T vs. D85N differential expression mRNA-protein enrichment analysis and key candidate gene related the gene expression analysis. (**a**) Differential expression mRNA-protein Gene Ontology (GO) term analysis; mRNA-protein, as classified into three main categories by the GO analysis, purple indicates the biological process, blue indicates the cellular component and green indicates the molecular function. (**b**) Differential expression mRNA-protein GO enrichment analysis. (**c**) Differential expression mRNA-protein KEGG analysis, in (**b**,**c**), the size and color of the bubble indicate the number and significant characters of the differential expression mRNA-proteins. (**d**) Differential expression mRNA-protein KEGG enrichment analysis and the orthologous signaling pathways are classified into different functions. (**e**) *CDK2*-related gene expression analysis, *p* < 0.05 (*), *p* < 0.01 (**) or *p* < 0.001 (***).

Finally, the GO analysis was performed with the DE genes from the integrated multi-omics analysis functional categories, including biological processes (BPs), cellular components (CCs) and molecular functions (MFs) for D85T vs. D85N, respectively. As Figure 4a shows, the annotated GO terms of the DAPs for the maternal dietary restriction nutrition comparable group covered protein phosphorylation, apoptotic process, positive regulation of transcription by RNA polymerase Ⅱ, protein transport, mRNA processing and positive regulation of cell proliferation of the BPs; nucleus, cytoplasm and extracellular exosome were the key subcategories of the CCs; nucleotide binding, DNA binding, zinc ion binding, calcium ion binding and ubiquitin protein ligase binding were the pivotal MF subcategories. The GO enrichment analysis indicated the GO terms with the function of protein phosphorylation, apoptotic process, cyclin E-CDK2 complex and nucleotide binding (Figure 4b). Furthermore, the results of the KEGG reveal that cellular senescence signaling pathways were significantly enriched (Figure 4c), and the KEGG enrichment analysis indicated that the cellular process, genetic information processing, organismal system and environmental information processing were major KEGG classifications, and cell growth and death, translation, endocrine system and signal transduction were key biological processes and regulation systems of the major KEGG classifications. At the same time, the *CDK2* gene with the functions of cell cycle regulation and myoblast differentiation and development was significantly enriched in the biological processes (Figure 4d). We found that the muscle growth and development marker genes *MYF5* and *MYOD* were down-regulated, and *SIX1*, *PAX7*, the cell cycle factors *CDK4*, *CDK6* and the *BCL-2* apoptosis factor were up-regulated to respond to the maternal dietary restriction (Figure 4e). 

### 3.5. Maternal Dietary Restriction Prevents Muscle Fiber Differentiation and Maturation at GD 135

#### 3.5.1. At the mRNA and Protein Levels, Genes Related to Muscle Fiber Protein Synthesis, Metabolism and Differentiation Change

**Figure 5:**
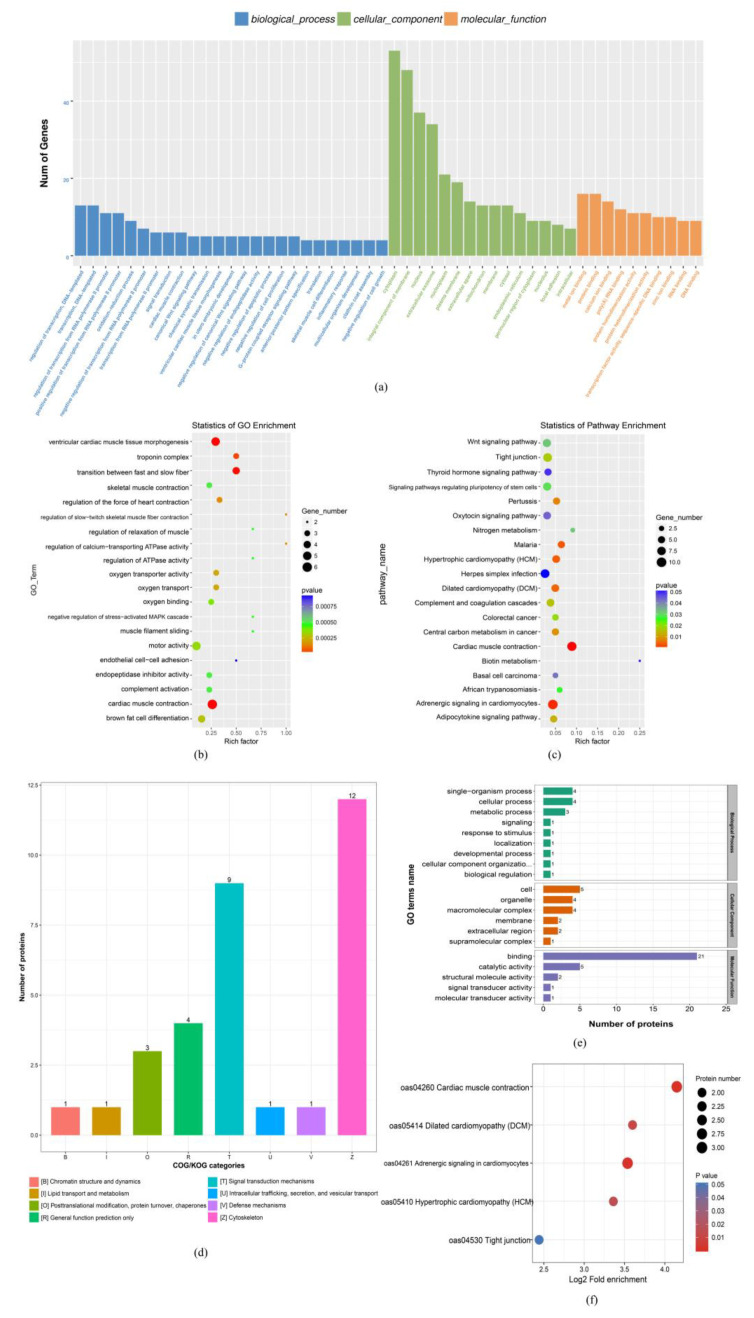
D135T vs. D135N differential expression mRNA and protein enrichment analysis. (**a**) Differential expression mRNA Gene Ontology (GO) term analysis; mRNAs are classified into three main categories by the GO analysis, blue indicates the biological process, green indicates the cellular component and orange indicates the molecular function. (**b**) Differential expression mRNA GO enrichment analysis. (**c**) Differential expression mRNA KEGG enrichment analysis, in (**b**,**c**), the size and color of the bubble indicate the number and significant characters of the differentially expressed mRNAs. (**d**) The functional classification chart of the differential abundance proteins (COG), the orthologous proteins were classified into different functions. (**e**) Differential protein abundance GO term analysis, the proteins were classified into three main categories by the GO analysis, green indicates a biological process, orange indicates the cellular component and purple indicates molecular function. (**f**) Differential protein abundance KEGG enrichment analysis, the size, and the color of the bubble indicate the number and significant characters of the differentially expressed proteins.

First, the GO analysis was performed with the mRNA functional categories, including biological processes (BPs), cellular components (CCs) and molecular functions (MFs) for D135T vs. D135N DE-mRNA, respectively. As Figure 5a shows, the annotated GO terms of DE-mRNA in the maternal dietary restriction comparable group cover the oxidation reduction process, negative regulation of endopeptidase activity, negative regulation of apoptotic process, negative regulation of cell proliferation, negative regulation of skeletal muscle cell differentiation, negative regulation of cell growth and multi cellular organism development as major subcategories of the BPs; cytoplasm, integral component of the membrane, nucleus, extracellular exosome and nucleoplasm were the key subcategories of the CCs; metal ion binding, protein binding, poly (A) RNA binding and calcium ion binding were the pivotal MF subcategories, and the GO enrichment analysis indicated that the GO terms with the function of transition between fast and slow fiber, regulation of slow-twitch skeletal muscle fiber contraction, skeletal muscle contraction and regulation of ATPase activity (Figure 5b). Furthermore, the results of the KEGG enrichment showed that the Wnt signaling pathways were significantly enriched, and the *MYOG* gene with the function of myoblast differentiation and development was significantly down-regulated in this signaling pathway (Figure 5c).

Then, the differential abundance proteins (DAPs) in D135T vs. D135N were analyzed by using the methods of the functional classification chart of the differentially expressed proteins (COG) and gene ontology (GO) analysis. As Figure 5d shows, the DAPs are mainly enriched in the major function classifications, such as chromatin structure and dynamics, post-translational modification, protein turnover chaperones, signal transduction mechanisms and cytoskeleton. Meanwhile, they were significantly enriched in 20 GO terms that play a molecular binding role in cellular biological processes (Figure 5e). The KEGG analysis of the DAPs found that all of the DAPs were significantly enriched in cardiac muscle contraction, dilated cardiomyopathy (DCM), adrenergic signaling in the cardiomyocytes and the hypertrophic cardiomyopathy (HCM) signaling pathway (Figure 5f). The DAP’s TNNC1 was significantly down-regulated in this comparison group.

#### 3.5.2. Under the Maternal Dietary Restriction, *TEAD1* Regulating the *VGLL3* Gene May Be a Potential Reason for Affecting the Myofiber Differentiation and Maturation

**Figure 6:**
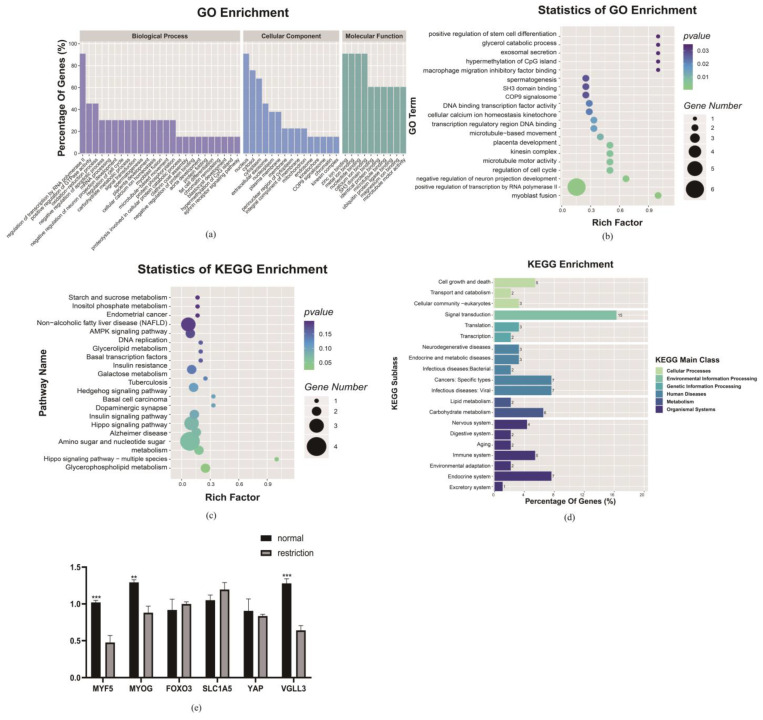
D135T vs. D135N differential expression mRNA-protein enrichment analysis and key candidate gene-related gene expression analysis. (**a**) Differential expression mRNA-protein Gene Ontology (GO) term analysis; mRNA-protein are classified into three main categories by the GO analysis, purple indicates the biological process, blue indicates the cellular component and green indicates the molecular function. (**b**) Differential expression mRNA-protein GO enrichment analysis. (**c**) Differential expression mRNA-protein KEGG analysis, in (**b**,**c**), the size and color of the bubble indicate the number and significant characters of the differentially expressed mRNA-proteins. (**d**) Differential expression mRNA-protein KEGG enrichment analysis, the orthologous signaling pathways are classified into different functions. (**e**) *TEAD1*-related gene expression analysis, *p* < 0.01 (**) or *p* < 0.001 (***).

Finally, the GO analysis was performed with the DE genes from the integrated multi-omics analysis functional categories, including biological processes (BPs), cellular components (CCs) and molecular functions (MFs) for D135T vs. D135N, respectively, as shown in Figure 6a. The annotated GO terms of the DAPs in the maternal dietary restriction nutrition comparable groups covered the positive regulation of transcription by RNA polymerase II, positive regulation of GTPase activity, oxidation-reduction process and negative regulation of the apoptotic process, protein transport, mRNA processing and positive regulation of cell proliferation of the BPs; nucleus, cytoplasm, cytosol and extracellular exosome were the key subcategories of the CCs; nucleotide binding, zinc ion binding, calcium ion binding and meta ion binding were the pivotal MF subcategories. The GO enrichment analysis indicated the GO terms with the function of positive regulation of stem cell differentiation, regulation of the cell cycle, and myoblast fusion (Figure 6b). Furthermore, the DE gene KEGG analysis revealed that the Hippo signaling pathway was significantly enriched (Figure 6c), and the gene with the function of regulating cell proliferation and differentiation, *TEAD1*, was a potential key regulatory factor for the significant enrichment in the Hippo signaling pathway. Furthermore, the KEGG enrichment analysis indicated that the cellular processing, genetic information processing, organismal system, metabolism and environmental information processing were major KEGG classifications, and the cell growth and death, translation, transcription, endocrine system, lipid metabolism and signal transduction were key biological processes and regulation systems in them (Figure 6d). Additionally, we found that the expression levels of the *TEAD1* transcription target factor *SLC1A5*, the downstream target gene *FoXO3* and the binding factor gene *YAP* were not significantly regulated, while the expression levels of the *TEAD1* binding factor gene *VGLL3* and muscle growth and development markers *MYF5* and *MYOG* were significantly down-regulated. All results may reveal that there may exist muscle fiber maldevelopment of the lamb in the maternal dietary restriction condition (Figure 6e).

### 3.6. mRNA Sequencing and Quantitative Protein Validation

In D135T vs. D135N, 18 proteins were validated using PRM, and 13 of those were quantified with two unique peptides. Following the analyses, the results demonstrated that the quantitation ratios of TMT and those of PRM were consistent with each other (Figure S2a). Therefore, the reliability of the TMT quantitation data and the advantages of the PRM technology were notable. The expression level of several genes were detected by qRT-PCR to validate the accuracy of the RNA sequencing data. As shown in Figure S2b, all of the results of the qRT-PCR were consistent with the sequencing data.

### 3.7. CDK2 Knockdown Affects the Cell Cycle and Proliferation of Sheep Primary Embryonic Myoblasts 

**Figure 7:**
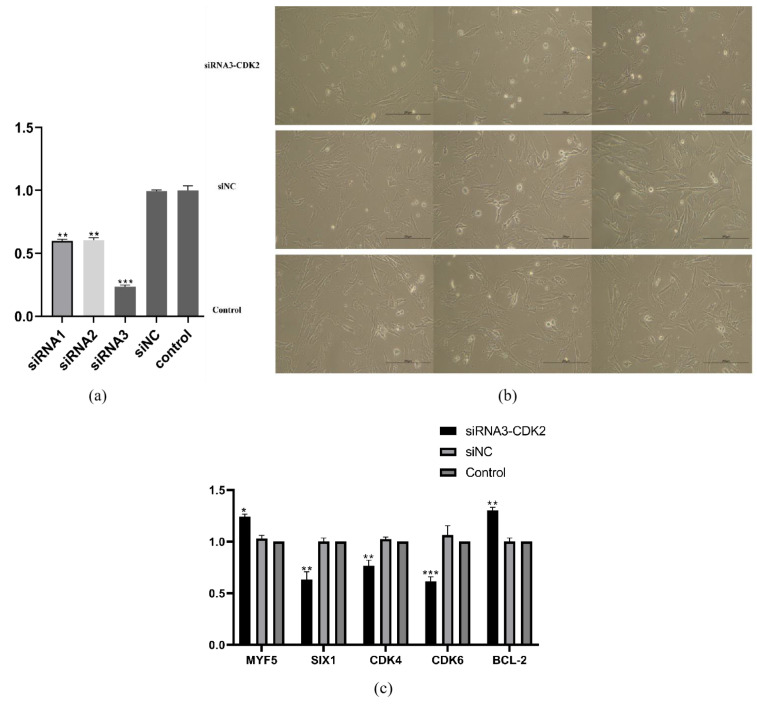
*CDK2* knockdown expression affects the cell cycle of sheep primary embryonic myoblasts. (**a**) Comparing the transfection effect of three siRNAs, (**b**) the phenotypes of the sheep primary embryonic myoblasts transfected with siRNA3-*CDK2,* siNC, and control were analyzed, and (**c**) the qRT-PCR results of the related genes after the siRNA3-*CDK2* knockdown, muscle growth and development, cell cycle, and apoptosis-related genes, *p <* 0.05 (*), *p <* 0.01 (**), or *p <* 0.001 (***).

Three pairs of *CDK2* siRNAs were constructed and transfected into sheep primary embryonic myoblasts, and it was found that the knockdown efficiency of siRNA3 was better, which could reduce the expression of the *CDK2* gene to about 23.4% of the original, and it had a lower efficiency (Figure 7a). At the same time, we found that the number of myoblasts in the *CDK2* knockdown group was less than that in siNC and the control group (Figure 7b). When the *CDK2* gene was knocked down, *CDK4*, *CDK6* and *SIX1* genes were significantly down-regulated and *MYF5* and *BCL-2* were significantly up-regulated, these results were identical to our integrated analysis (Figure 7c).

### 3.8. Low Nutrition Effect on the Sheep Primary Embryonic Myoblasts

**Figure 8:**
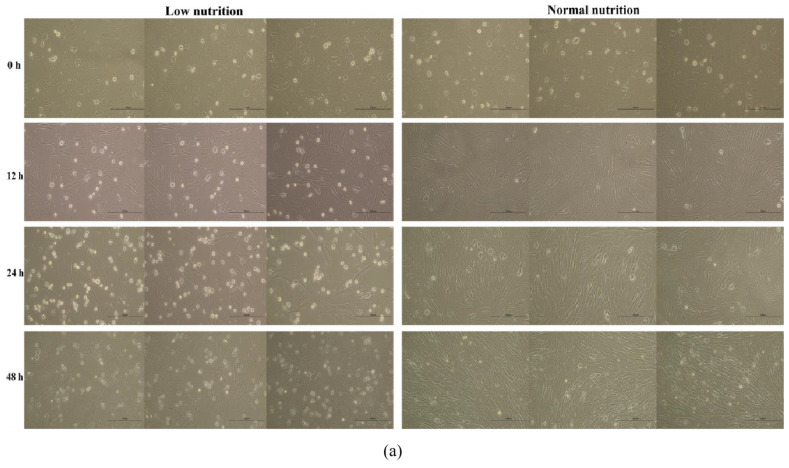

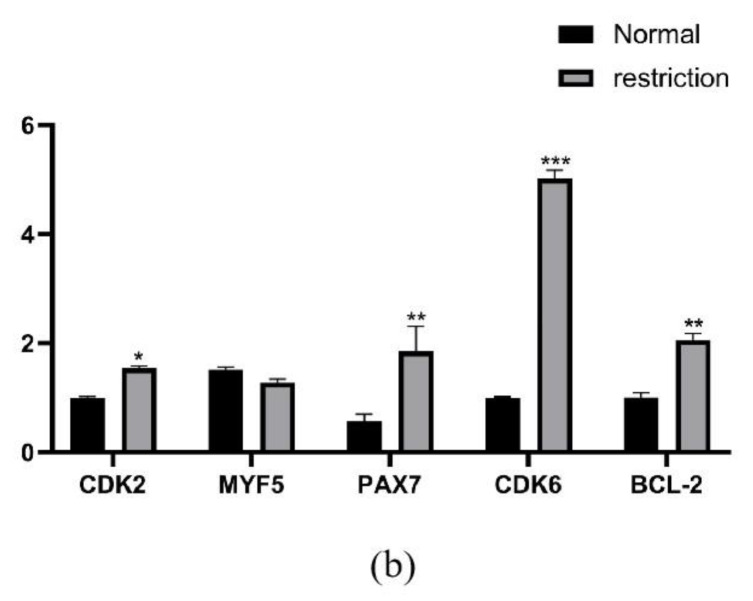
Low nutrient culture affects the sheep primary embryonic myoblast proliferation and growth. (**a**) The different period phenotypes of sheep primary embryonic myoblasts cultured with DMEM/F12 (low nutrition) and DMEM/F12 containing 10% FBS (normal nutrition). (**b**) qRT-PCR results of the related genes with the two experimental groups, *p* < 0.05 (*), *p* < 0.01 (**), or *p* < 0.001 (***).

The fusion of the primary sheep myoblasts was recorded as 0 h at 80%–90% and photographed. Then, the walled cells were then divided into restricted and normal nutrition groups for culture. At 12 h of incubation, the number of walled cells in the low-nutrition group was less than that in the normal-nutrition group. In contrast, the number of suspended cells was more than that in the normal nutrition group. At 24 h of continuous incubation, the number of walled cells in the low-nutrition group was significantly lower than that in the normal-nutrition group, while the number of suspended cells was significantly higher than that in the normal-nutrition group. Both groups of cells were continuously cultured for 48 h. In the normal nutrition group, the fusion of myoblasts reached 100%, and the morphology was uniform and evident. However, the number of walled myoblasts in the low-nutrition group did not show any significant difference after 24 h. Nevertheless, it was significantly lower than the normal nutrition group. Interestingly, the number of suspended cells in the low-nutrition group decreased after 48 h, the dead cells were present in the medium, and the morphology of the suspended cells differed from those in the normal nutrition group (Figure 8a). 

In addition, the results of the qRT-PCR revealed that the cell cycle genes *CDK2* and *CDK6* and the apoptosis gene *BCL-2* were significantly up-regulated in the cells of the low nutrition group, the myoblast proliferation gene *PAX7* was significantly up-regulated, and the expression of *MYF5*, a gene regulating myoblast differentiation, was lower than that of the normal nutrition group. This result was the same as our hypothetical conclusion in the integrated analysis (Figure 8b). 

## 4. Discussion

Numerous studies have shown that maternal diet affects the growth and development of embryonic organs in both humans and livestock [2,27,31]. Particularly, throughout the gestational stages of skeletal muscle growth and development, the rate of muscle fiber growth differs depending on whether the mother receives adequate, excessive or inadequate nourishment [33,35,42,43]. According to the early research, the aberrant maternal diet during pregnancy affected the fiber number, cross-sectional area, types, metabolism, insulin tolerance, gene expression and epigenetic modification of the skeletal muscle [20,25,39,44,45,46]. The potential molecule mechanism has been the subject of preliminary research. Embryonic skeletal muscle growth and development are influenced by maternal nutrition, however it is unclear what biochemical processes exist in this special maternal internal gestation environment. Sheep have been regarded as the best animal model to study the intrauterine condition, fetal development and newborn fate [16,17,18]. The influence of the mother’s prenatal nutrition on fetal growth and development in humans can be understood best by examining the molecular mechanism of maternal nutrition affecting fetal growth and development using a sheep model.

In this study, a maternal dietary restriction model was established, and expected that the system elaborates on the unclear genetic mechanism. It is well known that fetal skeletal muscle growth and development are complex biological processes. At early gestation, induction of the genes *Samd4*, *PKC*, *FoxO1*, *Wnt1*, *GH* and Ca^2+^ activates the myoblast fusion into myotubes. Then, myotubes grow into mature muscle fibers under the regulation and activation of various factors, such as *MRF4*, *MyHC* and *MCK*, at the late gestation stage [47]. However, at GD 85, there was no significant difference in the growth traits of fetal lambs, and the results of our integrated transcriptome and proteome analyses found that, compared with normal maternal nutrition, the growth and cell cycle of embryonic myoblasts were changed under maternal feeding restriction, and in this condition, biological processes, such as cell apoptosis and the cyclin E-CDK2 complex were active. The cell cycle regulation factor *CDK2* was up-regulated in the cellular senescence signaling pathway. Studies have shown that *CDK2* activity correlates with skeletal muscle differentiation, and the activity of *CDK2* is inhibited during the differentiation process [48]. As we all know, the terminal differentiation of skeletal muscle cells is normally accompanied by a withdrawal of the cells from active proliferation into a differentiation state, and this biological process has been correlated with a drastic decrease in *CDK2* activity [49,50]. Moreover, our findings suggest that maternal dietary restriction prevents the differentiation of fetal skeletal muscle cells and the formation of embryonic muscle fiber. At the same time, our assumption may be verified by the results of the up-regulation of *CDK2* to suppress cellular senescence [51]. Abnormal centrosome replication and regulation may be an influential cause of abnormal embryonic development in vitro [52]. *CDK2* activity is closely related to the centrosome replication cycle, and centrosome replication can be blocked by the protein repressor of *CDK2*, P21, as well as by antibodies to *CDK2*, while excess *CDK2* can restore centrosome replication [53,54]. P53 elevates the transcriptional level of P21 and inhibits the activity of *CDK2*, thereby inhibiting the multiple replications of centrosomes in a cell cycle [55]. It follows that the high *CDK2* expression inhibits P53 and P21 activity and promotes the replication of myocyte centrosomes. In contrast, *Gadd45* is a P53-dependent stress-inducible gene, and *Gadd45* is involved in the regulation of cell differentiation [56], and reduced P53 activity may have contributed to the reduced differentiation ability of myoblasts. The aforementioned results once again imply that maternal dietary restriction may increase *CDK2* gene expression, affecting the cycle and proliferation of myoblasts while impeding their differentiation and maturation. Additionally, a report has found that ablation of the CDK inhibitor can result in increased apoptosis and delay in the differentiation [57]. In our study, knocking down the *CDK2* expression by siRNA inhibited the proliferation of primary embryonic myoblasts of sheep and affected the expression of cell cycle factors and apoptosis factors. The results reached the same conclusion as previous function studies of the *CDK2* gene.

Meanwhile, the analysis of mRNA and proteins has similar results to our integration analysis. At the mRNA level, the expression of *ING2* was down-regulated, the biological processes of the negative regulation of cell proliferation, protein ubiquitination, negative regulation of signal translation, apoptosis process and negative regulation of cell growth were activated, and the GO terms of regulation of the spindle assembly, regulation of the kinetochore assembly, limb development and negative regulation of the translation were enriched. *ING2*, as a critical regulator of chromatin remodeling, it controls gene expression and has been implicated in the regulation of cell proliferation, death and muscle differentiation [58]. It is involved in cell cycle regulation, DNA damage response, apoptosis and senescence [59]. Meanwhile, this down-regulated gene can disrupt the cell cycle and block muscle differentiation [58]. Interestingly, *ING2* can strongly enhance the transcriptional-transactivation activity of p53 to negatively regulate cell proliferation, and centrosome replication is inhibited during the cell cycle by p53 negatively regulating the *CDK2* activity [60], and the centrosome replication is inhibited during the cell cycle by p53 negatively regulating the *CDK2* activity [61]. It follows that down-regulated *ING2* reduces the expression of p53 and leads to *CDK2* up-regulation, which may be a potential reason for fetal skeletal muscle cell differentiation inhibition, but this speculation still needs to be tested in the future. In addition, at the protein level, FAU was significantly up-regulated in the maternal dietary restriction. This protein has been demonstrated to regulate apoptosis when it is expressed [62], and in this study, the results of the qRT-PCR showed that the expression of the cell cycle factors *CDK4* and *CDK6* and the *BCL-2* apoptosis factor were up-regulated, this conclusion is consistent with the results of the sequencing of the activation process of apoptosis. Meanwhile, the muscle growth and development marker genes *MYF5* and *MYOD* were significantly down-regulated, and *PAX7* and *SIX1* were up-regulated, those results all showed that the differentiation ability of fetal myoblast reduction and cell cycle changing in the maternal dietary restriction condition also validated our results. Both studies indicate that maternal nutritional restriction at GD 85 influences the cell cycle and prevents myoblast differentiation, the *CDK2* may be a critical gene of this biological process. As well, experiments on the proliferation and growth of sheep embryonic myoblasts at different nutritional levels revealed similar results to the integrated multi-omics findings of our present study: nutritional restriction altered the expression of genes related to the cell cycle, myoblast proliferation, and apoptosis, inhibited the proliferation of myogenic cells and caused their apoptosis.

While in D135T vs. D135N, we found that the body weight of the fetus decreased significantly, and the mRNA sequencing showed that negative regulation of the apoptotic process, negative regulation of cell proliferation, negative regulation of skeletal muscle cell differentiation and negative regulation of cell growth as major biological processes, are activated, GO terms with the function of transition between fast and slow fiber, regulation of slow-twitch skeletal muscle fiber contraction, skeletal muscle contraction and regulation are enriched in these comparable groups, and the maker gene of skeletal muscle differentiation *MYOG is* down-regulated in the WNT signaling pathway. All results show that maternal dietary deficiency influences fetal skeletal muscle cell growth and differentiation. At the same time, our study found that DAPs TNNC1 is significantly down-expressed as an 18 kDa member of the EF-hand Ca^2+^-binding protein family in both slow skeletal and cardiac tissue [63,64,65,66], and this gene has the regulation function of cardiomyopathies [67]. These DAPs are enriched myopathy-related signaling pathways in this study. The influence of maternal dietary restriction negatively regulated the fetal skeletal muscle growth and development, which may result in relative myopathy. 

*TEAD1*, a gene that promotes skeletal muscle proliferation and differentiation [68], plays a significant role in key biological processes, including stem cell differentiation, cell cycle and myoblast fusion. This gene is enriched in the Hippo signaling pathway in our transcriptome and proteome integrated analyses of D135T vs. D135N. Meanwhile, considerable studies showed that in the Hippo signaling pathway, *TEAD1* plays a crucial role in skeletal muscle growth, development and multiple embryonic contexts by activating gene expression [69,70,71,72]. *TEAD1* is required for cell growth by regulating the key factor YAP of the Hippo signaling pathway [73]. Moreover, *TEAD1* is also needed for regulating muscle growth and development by acting on the transcriptional target factor SLC1A5 and the downstream target gene *FoXO3* [69,71]. As well, our team found that *TEAD1* knockdown expression by siRNA inhibited the proliferation of sheep primary embryonic myoblasts. CCK8 experiments and scratch assay reached the same conclusion, the muscle marker genes (*MYF5*, *PAX7*, and *MYOD*), the transcription target factor SLC1A5 and the downstream target gene *FoXO3* were significantly down-regulated (data not shown). In this research, we found that the expression of *YAP*, SLC1A5 and *FoXO3* were not significantly expressed between the maternal dietary restriction and normal embryonic skeletal muscle samples, which may indicate that maternal dietary restriction during pregnancy cannot affect the *TEAD1* gene-related regulatory factors and gene expression. While the muscle growth and development marker genes *MYF5* and *MYOG* were down-regulated by the maternal dietary restriction at GD 135, and that resulted in myofiber differentiation and maturation being reduced in fetal sheep. From the results of the quantitative analysis of the TEAD1-related regulatory genes, we hypothesized that maternal dietary restriction may cause reduced myofiber differentiation and maturation as a result of *TEAD1* binding the significantly down-regulated gene, *VGLL3*. The Hippo signaling transduction network is involved in regulating development, cell proliferation and body size [74] and plays an important role in skeletal muscle growth and development [75]. Related studies have found that *YAP* and *TEAD1* have been closely related to muscle development and regenerative myogenesis [76,77,78]. There is a study that demonstrated that in the occurrence of dystrophin deficiency, the somatic stem cells are not poised to affect the immediate muscle tissue, yet the overexpression of *TEAD1* can ameliorate the pathology by increasing the number of satellite cells [79], and the *VGLL3* gene, in combination with the *TEAD1* gene, regulates the skeletal muscle myogenesis process [80]. The study findings indicate that the *TEAD1* gene binding to the down-regulated gene of *VGLL3* results in reduced myofiber differentiation and maturation, suggesting that the *TEAD1* gene may play a key role in this biological process. To fully comprehend the mechanism by which the *TEAD1* gene controls this process, more investigation is necessary. Based on the sheep gestation model we constructed, we analyzed the potential gene expression changes and regulation mechanisms in maternal dietary restriction conditions, compared with normal maternal nutritional conditions and maternal feeding restriction at GD 85. The results showed that maternal dietary restriction leads to the *CDK2* up-regulated expression and changes the cell cycle of the fetal skeletal muscle cells and apoptosis metabolism. The maternal dietary restriction at GD 135 impacts the development of embryonic muscles, and the *TEAD1* gene may be a factor in the fetal weight loss brought on by maternal malnutrition. These findings indicate that the candidate genes may have an impact on fetal growth and development through maternal diet.

## 5. Conclusions

Human health and fetal growth and development are intimately intertwined. We discovered that at GD 135 the fetal weight under the maternal dietary restriction was considerably lower than under normal circumstances based on the sheep gestation model we built. The multi-omics analysis reveals that maternal dietary restriction leads to the *CDK2* up-regulated expression and changes the cell cycle of fetal skeletal muscle cells and apoptosis metabolism at GD 85. The maternal dietary restriction at GD 135 impacts the development of the embryonic muscles, and the *TEAD1* gene may contribute to the maternal dietary restriction-induced fetal weight reduction. These findings suggest that these potential genes could play a significant role in the molecular regulatory mechanisms that control how maternal dietary restriction affects the growth and development of the fetus. The ability of primary embryonic myoblasts to proliferate could be inhibited by the *CDK2* function loss gene analysis, and the expression levels of cell cycle regulatory components were considerably altered. The number of myoblasts decreases and the expression of cell cycle and muscle differential marker factors changes in low-nutrient culture conditions. Using the sheep gestation model, we studied the molecular mechanisms of fetal muscle growth and development using multi-omics and molecular biology techniques. This study laid a strong foundation for understanding the molecular regulatory mechanisms of maternal and infant nutrition on fetal skeletal muscle growth and development as well as for the development of new medications and the treatment of associated metabolic diseases. 

## Data Availability

The raw reads of RNA samples are available on the NCBI GEO database Transcriptomic accession: GSE127287, “https://www.ncbi.nlm.nih.gov/geo/query/acc.cgi?acc=GSE127287 (accessed on 18 November 2019)”. All relevant data were uploaded to the iProX Integrated Proteome Resources Database Proteomic accession: IPX0001527000, “https://www.iprox.cn/page/PSV023.html;?url=1676635931680cAgm, password: cpMf (accessed on 17 February 2023)”.

## Supplementary Materials

The following supporting information can be downloaded at: https://www.mdpi.com/article/10.3390/nu15041051/s1, Figure S1: mRNA and protein expression profile analysis. (a) mRNA PCA analysis, PCA plots of the two components of all samples. The fraction of the total variance explained is reported on each axis between parentheses; (b) differential expression mRNAs volcano plot analysis at (a) D85T vs. D85N and (b) D135T vs. D135N, where the red points indicate the up-regulation of the differentially expressed mRNA, the blue points indicate the down-regulation of the differentially expressed mRNA, and the grey points indicate no difference. (c) Protein PCA analysis, PCA plots of the two components of all samples. The fraction of the total variance explained is reported on each axis between parentheses; (d) differential expression proteins volcano plot analysis at (a) D85T vs. D85N and (b) D135T vs. D135N, where the red points indicate the up-regulation of differentially expressed proteins, the blue points indicate the down-regulation of the differentially expressed proteins, and the grey points indicate no difference. (e) mRNA-protein correlation analysis of (a) D85T vs. D85N and (b) D135T vs. D135N, the red points indicate the differential expression of mRNA-protein, and the grey points indicate no difference; Figure S2: Quantitative protein and mRNA sequencing validation. (a) The ratio of TMT and PRM is quantitative, the orange indicates the TMT quantitative ratio in D135T vs. D135N, and the blue indicates the PRM quantitative ratio in D135T vs. D135N. (b) The polyline graph shows the FPKM value of the sequencing, and the histogram shows the relative quantitative results, a and b indicate a significant difference. Table S1: Standard for feeding full-price pellets to pregnant ewes in New Zealand; Table S2: Primers for the qRT-PCR; Table S3: PRM validation of the differentially abundant proteins; Table S4: Sequence of *CDK2* siRNAs; Table S5: The list of all mRNA-protein correlations.
