# Supplementary figures and images for: Multi-Omics Analysis Reveals the Potential Effects of Maternal Dietary Restriction on Fetal Muscle Growth and Development
Source: Nutrients. 2023 Feb 20;15(4):1051. doi: 10.3390/nu15041051 (PMC9964303; doi:10.3390/nu15041051)

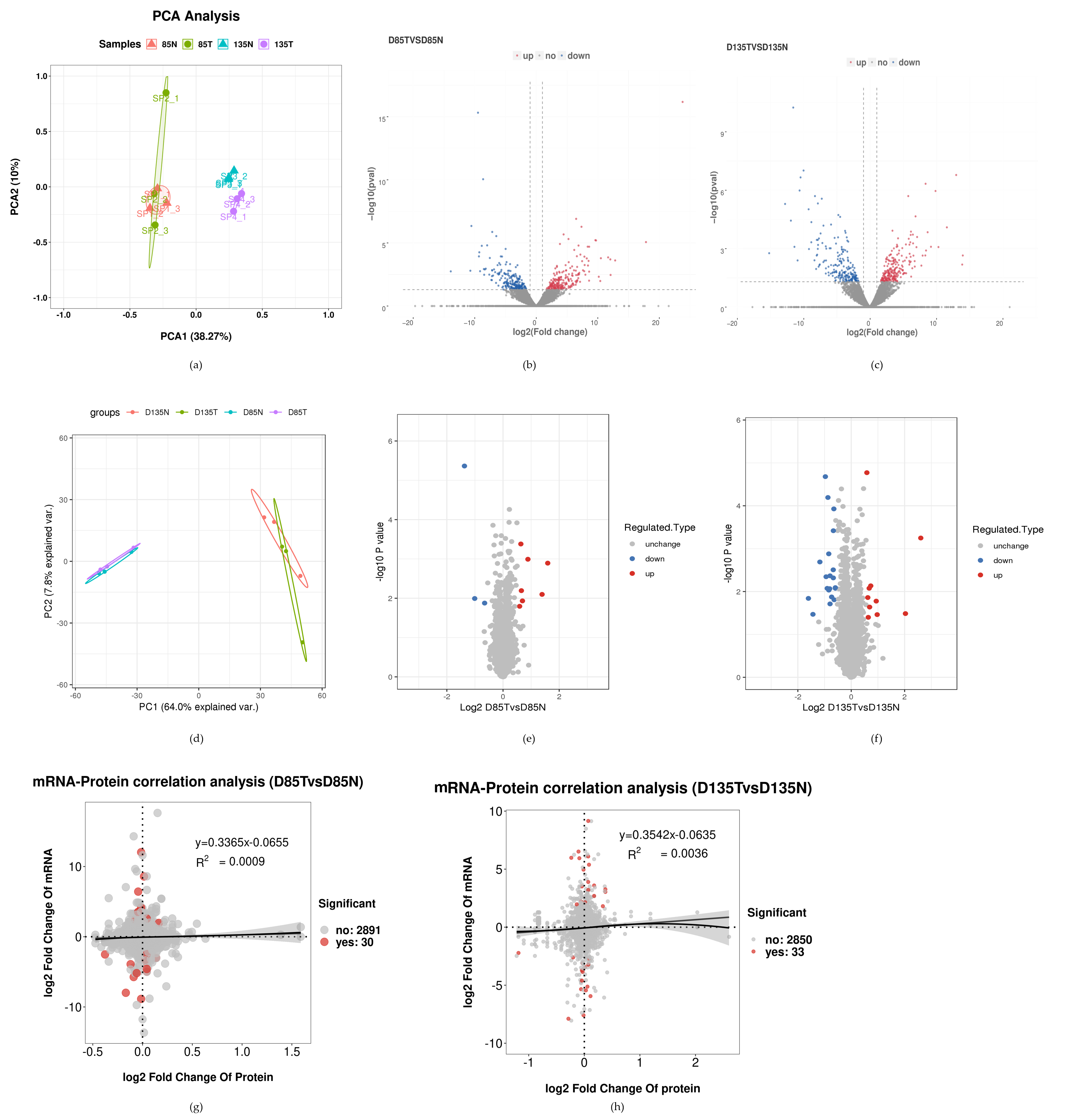

Supplement: Supplementary file 1 [file nutrients-15-01051-s001.zip › Figure S1.tif]

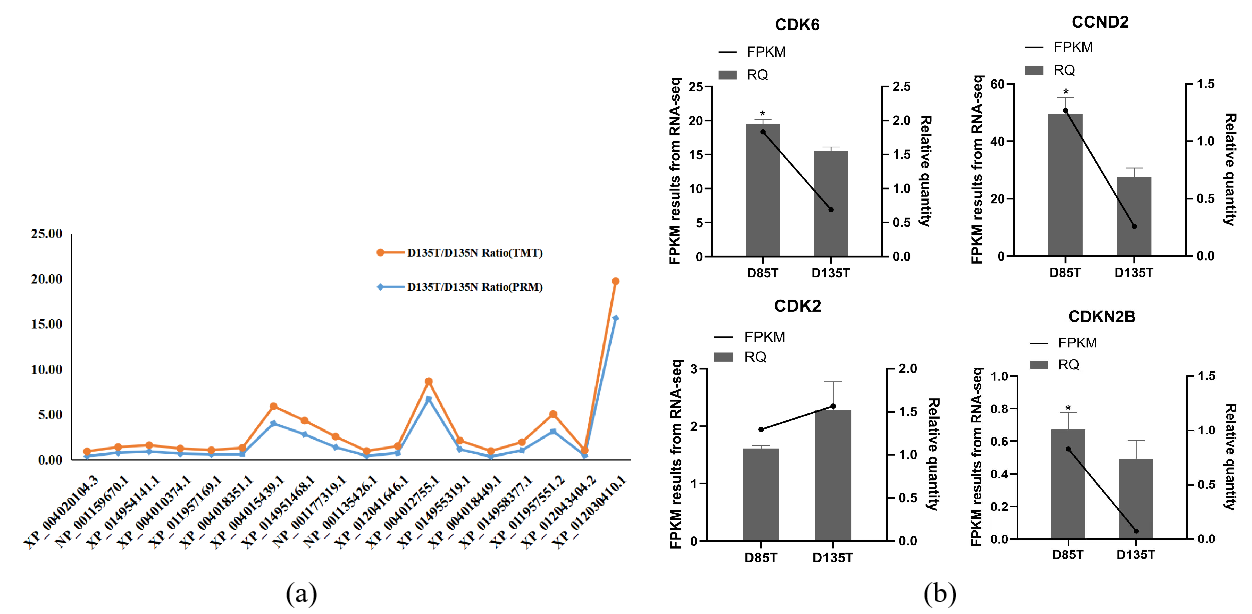

Supplement: Supplementary file 1 [file nutrients-15-01051-s001.zip › Figure S2.tif]
